# 1-[(Bromo­meth­yl)(phen­yl)meth­ylene]-2-(2,4-dinitro­phen­yl)hydrazine

**DOI:** 10.1107/S1600536809016225

**Published:** 2009-05-07

**Authors:** Abdussalam Salhin, Norfarhah Abdul Razak, I. A. Rahman

**Affiliations:** a School of Chemical Sciences, Universiti Sains Malaysia, USM, Penang, 11800, Malaysia

## Abstract

The title compound, C_14_H_11_BrN_4_O_4_, comprises two crystallographically independent mol­ecules (*A* and *B*) in the asymmetric unit. In mol­ecule *B*, intra­molecular bifurcated N—H⋯O and N—H⋯Br hydrogen bonds and in mol­ecule *A*, an intra­molecular N—H⋯O hydrogen bond generate *S*(6) ring motifs. The dihedral angle between the phenyl and benzene rings is 5.44 (6) in mol­ecule *A* and 7.63 (6)° in mol­ecule *B*. The *ortho*- and *meta*-nitro substituents make dihedral angles of 6.67 (15) and 2.26 (15)° to the attached benzene ring in mol­ecule *A* and 6.37 (17) and 5.81 (16)° in mol­ecule *B*. The Br atom in mol­ecule *B *is disordered over two positions with a refined site-occupancy ratio of 0.61 (3):0.39 (3). Inter­esting features of the crystal structure are the short Br⋯N [3.257 (3)–3.294 (4) Å], Br⋯O [3.279 (3)–3.307 (4) Å] and O⋯O [2.9319 (16)–2.9995 (16) Å] contacts, which are shorter than the sum of the van der Waals radii of these atoms. The crystal structure is further stabilized by inter­molecular C—H⋯O and π–π inter­actions [centroid–centroid distances = 3.6643 (8)–3.8514 (8) Å].

## Related literature

For bond-length data, see: Allen *et al.* (1987 ▶). For hydrogen-bond motifs, see: Bernstein *et al.* (1995 ▶). For related structures and bioactivity, see; for example: Salhin *et al.* (2007 ▶); Tameem *et al.* (2006 ▶, 2007 ▶, 2008 ▶); Rollas & Küçükgüzel (2007 ▶); Shao *et al.* (2008 ▶).
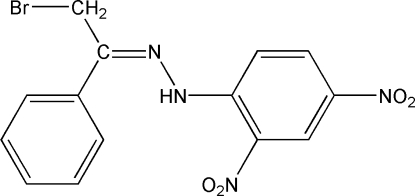


## Experimental

### 

#### Crystal data

C_14_H_11_BrN_4_O_4_
*M* *_r_* = 379.18
Monoclinic, 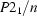

*a* = 13.0803 (3) Å
*b* = 15.3626 (3) Å
*c* = 14.1512 (2) Å
β = 91.903 (1)°
*V* = 2842.08 (9) Å^3^
*Z* = 8
Mo *K*α radiation
μ = 2.92 mm^−1^
*T* = 100 K
0.59 × 0.34 × 0.33 mm

#### Data collection

Bruker SMART APEXII CCD area-detector diffractometer
Absorption correction: multi-scan (**SADABS**; Bruker, 2005 ▶) *T*_min_ = 0.238, *T*_max_ = 0.381
45788 measured reflections
11112 independent reflections
8666 reflections with *I* > 2σ(*I*)
*R*_int_ = 0.031

#### Refinement

*R*[*F* ^2^ > 2σ(*F* ^2^)] = 0.031
*wR*(*F* ^2^) = 0.077
*S* = 1.01
11112 reflections
433 parameters
H atoms treated by a mixture of independent and constrained refinement
Δρ_max_ = 0.69 e Å^−3^
Δρ_min_ = −0.35 e Å^−3^

### 

Data collection: *APEX2* (Bruker, 2005 ▶); cell refinement: *SAINT* (Bruker, 2005 ▶); data reduction: *SAINT*; program(s) used to solve structure: *SHELXTL* (Sheldrick, 2008 ▶); program(s) used to refine structure: *SHELXTL*; molecular graphics: *SHELXTL*; software used to prepare material for publication: *SHELXTL* and *PLATON* (Spek, 2009 ▶).

## Supplementary Material

Crystal structure: contains datablocks global, I. DOI: 10.1107/S1600536809016225/at2763sup1.cif

Structure factors: contains datablocks I. DOI: 10.1107/S1600536809016225/at2763Isup2.hkl

Additional supplementary materials:  crystallographic information; 3D view; checkCIF report

## Figures and Tables

**Table 1:** Hydrogen-bond geometry (Å, °)

*D*—H⋯*A*	*D*—H	H⋯*A*	*D*⋯*A*	*D*—H⋯*A*
N2*B*—H1*NB*⋯Br1*B*	0.82 (2)	2.826 (19)	3.3764 (12)	126.2 (15)
N2*B*—H1*NB*⋯O1*B*	0.82 (2)	1.969 (19)	2.6159 (16)	135.1 (17)
N2*A*—H1*NA*⋯O1*A*	0.79 (2)	2.02 (2)	2.6120 (16)	131.8 (19)
C2*B*—H2*BA*⋯Br1*A*^i^	0.93	2.93	3.673 (3)	138
C14*B*—H14*C*⋯O1*A*^i^	0.97	2.49	3.3352 (17)	145
C14*B*—H14*D*⋯O3*A*^ii^	0.97	2.52	3.3745 (18)	147

Symmetry codes: (i) 
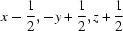
; (ii) 
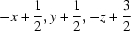
.

**Fig. 1.**
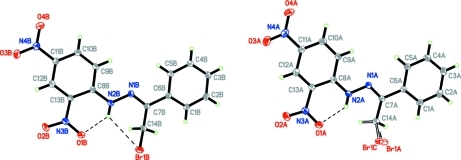
The molecular structure of the title compound, showing 30% probability displacement ellipsoids and the atomic numbering.

**Fig. 2.**
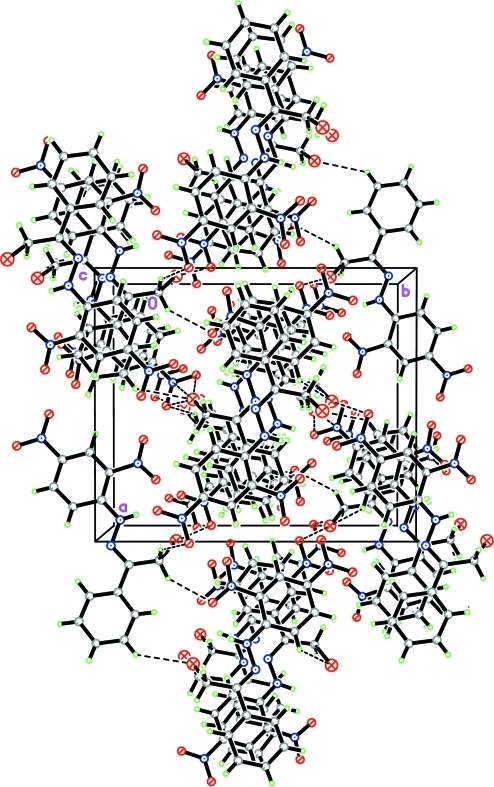
The crystal packing of the title compound, viewed down the c-axis showing intermolecular interactions. Intermolecular interactions are shown as dashed lines.

## supplementary crystallographic information

### Comment

In view of the importance of hydrazone derivatives in chemical and biological applications (Rollas & Küçükgüzel, 2007; Shao *et al.*, 2008), a series of hydrazone derivatives has been prepared in our laboratory, from which several X-ray structures have been reported (Salhin *et al.*, 2007; Tameem *et al.*, 2006, 2007 and 2008).

The title compound, C_14_H_11_BrN_4_O_4_, comprises two crystallographically independent molecules (A, B) in the asymmetric unit. In molecule B, intramolecular bifurcated N—H···O and N—H···Br hydrogen bonds and in molecule A, an intramolecular N—H···O hydrogen bond generate *S(6)* ring motifs. The dihedral angle between the phenyl rings in molecules A and B are 5.44 (6) and 7.63 (6)°, respectively. The *ortho* and *meta* nitro-substituents make the dihedral angles of 6.67 (15) and 2.26 (15)° in molecule A and 6.37917) and 5.81 (16)° to the benzene ring they are attached. The bromine group was disordered over two positins with a refined site-occupancy ratio of 0.61 (3)/0.39 (3) in molecule B. The crystal structure is further stabilized by intermolecular C—H···O and π-π [*Cg*1··· *Cg*1^iii^ = 3.6643 (8) Å, (iii) 2 - x, -y, 1 - z; *Cg*1··· *Cg*4^iv^ = 3.7308 (8) Å, (iv) 1 - x, -y, 1 - z; *Cg*1···*Cg*2^iv^ = 3.7013 (8) Å; *Cg*2···*Cg*3^iv^ = 3.7012 (8) Å; *Cg*3···*Cg*4^v^ = 3.8514 (8) Å, (v) -x, -y, 2 - z: *Cg*1, *Cg*2, *Cg*3, *Cg*4 are the centroids of the C1A–C6A, C8A–C13A, C1B–C6B, and C8B–C13b rings].

The interesting features of the crystal structure are short Br1A···N4A^vi^ [3.257 (3), (vi) 3/2-x,1/2+y,1/2-z], Br1C···N4A^vi^ [3.294 (4) Å], Br1A···O3A^viii^ [3.279 (3), (viii) 3/2-x,-1/2+y,1/2-z], Br1C···O3A^vii^ [3.307 (4) Å], O2A···O3A^iv^ [2.9319 (16) Å], and O1A···O4B^vii^ [2.9995(160 Å, (vii) 1/2-x,1/2+y,3/2-z] contacts which are shorter than the sum of the van der Waals radii of these atoms.

### Experimental

2,4-Dinitrophenylhydrazine (0.5 g, 2.5 mmol) was dissolved in ethanol (10 ml), and H_2_SO_4_ solution (98%, 1 ml) was added slowly with stirring. The solution was heated on a water bath for several minutes until it cleared. An ethanol solution (5 ml) of α-bromoacetophenone (0.5 g, 2.5 mmol) was dropped slowly into the above solution with continuous stirring. The mixture was heated for another 5 minutes on water bath. On cooling to room temperature, an orange precipitate was formed. Recrystallization from ethanol solution produced the crystals of the title compound.

### Refinement

The N-bound hydrogen atoms were located from the difference Fourier map and refined freely, see Table 1. The rest of the H atoms were positioned geometrically and refined as riding model with C—H = 0.93–0.97 and *U*_iso_(H) = 1.2 *U*_eq_(C).

### Crystal data

**Table:**

C_14_H_11_BrN_4_O_4_	*F*(000) = 1520
*M**_r_* = 379.18	*D*_x_ = 1.772 Mg m^−^^3^
Monoclinic, *P*2_1_/*n*	Mo *K*α radiation, λ = 0.71073 Å
Hall symbol: -P 2yn	Cell parameters from 9995 reflections
*a* = 13.0803 (3) Å	θ = 2.2–33.8°
*b* = 15.3626 (3) Å	µ = 2.92 mm^−^^1^
*c* = 14.1512 (2) Å	*T* = 100 K
β = 91.903 (1)°	Block, orange
*V* = 2842.08 (9) Å^3^	0.59 × 0.34 × 0.33 mm
*Z* = 8	

### Data collection

**Table:**

Bruker SMART APEXII CCD area-detector diffractometer	11112 independent reflections
Radiation source: fine-focus sealed tube	8666 reflections with *I* > 2σ(*I*)
graphite	*R*_int_ = 0.031
φ and ω scans	θ_max_ = 33.5°, θ_min_ = 2.0°
Absorption correction: multi-scan (*SADABS*; Bruker, 2005)	*h* = −19→20
*T*_min_ = 0.238, *T*_max_ = 0.381	*k* = −23→20
45788 measured reflections	*l* = −21→21

### Refinement

**Table:**

Refinement on *F*^2^	Primary atom site location: structure-invariant direct methods
Least-squares matrix: full	Secondary atom site location: difference Fourier map
*R*[*F*^2^ > 2σ(*F*^2^)] = 0.031	Hydrogen site location: inferred from neighbouring sites
*wR*(*F*^2^) = 0.077	H atoms treated by a mixture of independent and constrained refinement
*S* = 1.01	*w* = 1/[σ^2^(*F*_o_^2^) + (0.0364*P*)^2^ + 1.0321*P*] where *P* = (*F*_o_^2^ + 2*F*_c_^2^)/3
11112 reflections	(Δ/σ)_max_ = 0.007
433 parameters	Δρ_max_ = 0.69 e Å^−^^3^
0 restraints	Δρ_min_ = −0.34 e Å^−^^3^

### Special details

**Table:**

Experimental. The low-temperature data was collected with the Oxford Cyrosystem Cobra low-temperature attachment.
Geometry. All e.s.d.'s (except the e.s.d. in the dihedral angle between two l.s. planes) are estimated using the full covariance matrix. The cell e.s.d.'s are taken into account individually in the estimation of e.s.d.'s in distances, angles and torsion angles; correlations between e.s.d.'s in cell parameters are only used when they are defined by crystal symmetry. An approximate (isotropic) treatment of cell e.s.d.'s is used for estimating e.s.d.'s involving l.s. planes.
Refinement. Refinement of *F*^2^ against ALL reflections. The weighted *R*-factor *wR* and goodness of fit *S* are based on *F*^2^, conventional *R*-factors *R* are based on *F*, with *F* set to zero for negative *F*^2^. The threshold expression of *F*^2^ > σ(*F*^2^) is used only for calculating *R*-factors(gt) *etc*. and is not relevant to the choice of reflections for refinement. *R*-factors based on *F*^2^ are statistically about twice as large as those based on *F*, and *R*- factors based on ALL data will be even larger.

### Fractional atomic coordinates and isotropic or equivalent isotropic displacement parameters (Å^2^)

**Table:**

	*x*	*y*	*z*	*U*_iso_*/*U*_eq_	Occ. (<1)
Br1C	0.9686 (2)	0.1986 (2)	0.2704 (3)	0.0201 (6)	0.39 (3)
Br1A	0.9789 (4)	0.20086 (17)	0.2662 (2)	0.0351 (3)	0.61 (3)
O1A	0.73417 (8)	0.13615 (7)	0.39559 (8)	0.0233 (2)	
O2A	0.57980 (8)	0.08996 (7)	0.41372 (8)	0.0265 (2)	
O3A	0.49748 (9)	−0.20591 (8)	0.44038 (8)	0.0278 (2)	
O4A	0.61595 (9)	−0.30399 (7)	0.45462 (8)	0.0279 (2)	
N1A	0.98576 (9)	0.00033 (8)	0.38344 (8)	0.0160 (2)	
N2A	0.88776 (9)	0.02751 (8)	0.39522 (8)	0.0169 (2)	
N3A	0.67186 (9)	0.07666 (8)	0.40818 (8)	0.0184 (2)	
N4A	0.58779 (10)	−0.22801 (8)	0.44372 (8)	0.0214 (2)	
C1A	1.23883 (10)	0.08672 (9)	0.33786 (9)	0.0182 (2)	
H1AA	1.2251	0.1460	0.3329	0.022*	
C2A	1.33726 (11)	0.05613 (10)	0.32338 (10)	0.0207 (3)	
H2AA	1.3890	0.0951	0.3094	0.025*	
C3A	1.35843 (11)	−0.03208 (10)	0.32978 (10)	0.0210 (3)	
H3AA	1.4242	−0.0524	0.3199	0.025*	
C4A	1.28092 (11)	−0.09024 (10)	0.35096 (10)	0.0204 (3)	
H4AA	1.2951	−0.1495	0.3553	0.024*	
C5A	1.18284 (10)	−0.06035 (9)	0.36562 (9)	0.0177 (2)	
H5AA	1.1314	−0.0997	0.3794	0.021*	
C6A	1.16034 (10)	0.02883 (8)	0.35980 (9)	0.0149 (2)	
C7A	1.05537 (10)	0.05985 (8)	0.37716 (9)	0.0154 (2)	
C8A	0.81403 (10)	−0.03332 (8)	0.40698 (9)	0.0155 (2)	
C9A	0.84032 (10)	−0.12258 (9)	0.41293 (9)	0.0171 (2)	
H9AA	0.9084	−0.1388	0.4081	0.021*	
C10A	0.76784 (11)	−0.18574 (9)	0.42569 (9)	0.0188 (2)	
H10A	0.7866	−0.2441	0.4294	0.023*	
C11A	0.66562 (11)	−0.16139 (9)	0.43304 (9)	0.0179 (2)	
C12A	0.63592 (10)	−0.07562 (9)	0.42875 (9)	0.0177 (2)	
H12A	0.5676	−0.0605	0.4348	0.021*	
C13A	0.70905 (10)	−0.01200 (9)	0.41530 (9)	0.0163 (2)	
C14A	1.03381 (11)	0.15508 (9)	0.38699 (9)	0.0179 (2)	
H14A	0.9897	0.1645	0.4389	0.022*	0.61 (3)
H14B	1.0965	0.1860	0.3998	0.022*	0.61 (3)
H14E	1.0957	0.1854	0.4049	0.022*	0.39 (3)
H14F	0.9853	0.1641	0.4355	0.022*	0.39 (3)
Br1B	0.013792 (12)	0.244826 (9)	0.748873 (10)	0.02245 (4)	
O1B	−0.22221 (8)	0.18339 (7)	0.85993 (8)	0.0230 (2)	
O2B	−0.37193 (9)	0.14659 (8)	0.90464 (11)	0.0387 (3)	
O3B	−0.46689 (8)	−0.14664 (8)	0.95307 (9)	0.0301 (2)	
O4B	−0.35034 (9)	−0.24598 (7)	0.97032 (9)	0.0286 (2)	
N1B	0.02449 (9)	0.04055 (8)	0.85746 (8)	0.0164 (2)	
N2B	−0.07206 (9)	0.07090 (8)	0.86865 (8)	0.0172 (2)	
N3B	−0.28334 (9)	0.12826 (8)	0.88791 (9)	0.0201 (2)	
N4B	−0.37744 (9)	−0.17037 (8)	0.95376 (8)	0.0200 (2)	
C1B	0.28568 (10)	0.11582 (9)	0.82555 (9)	0.0181 (2)	
H1BA	0.2773	0.1759	0.8236	0.022*	
C2B	0.38267 (10)	0.07996 (9)	0.81648 (10)	0.0202 (3)	
H2BA	0.4387	0.1163	0.8091	0.024*	
C3B	0.39609 (11)	−0.00974 (10)	0.81839 (10)	0.0202 (3)	
H3BA	0.4609	−0.0336	0.8124	0.024*	
C4B	0.31187 (11)	−0.06353 (9)	0.82929 (10)	0.0211 (3)	
H4BA	0.3204	−0.1236	0.8300	0.025*	
C5B	0.21560 (11)	−0.02835 (9)	0.83908 (10)	0.0189 (3)	
H5BA	0.1599	−0.0651	0.8467	0.023*	
C6B	0.20080 (10)	0.06198 (8)	0.83760 (9)	0.0150 (2)	
C7B	0.09677 (10)	0.09733 (8)	0.85066 (9)	0.0153 (2)	
C8B	−0.14694 (10)	0.01355 (8)	0.88991 (9)	0.0151 (2)	
C9B	−0.12331 (10)	−0.07567 (9)	0.90438 (9)	0.0169 (2)	
H9BA	−0.0560	−0.0943	0.9000	0.020*	
C10B	−0.19764 (10)	−0.13504 (9)	0.92470 (9)	0.0176 (2)	
H10B	−0.1811	−0.1935	0.9329	0.021*	
C11B	−0.29861 (10)	−0.10694 (9)	0.93296 (9)	0.0165 (2)	
C12B	−0.32537 (10)	−0.02108 (9)	0.92251 (9)	0.0169 (2)	
H12B	−0.3926	−0.0033	0.9301	0.020*	
C13B	−0.25005 (10)	0.03894 (9)	0.90035 (9)	0.0158 (2)	
C14B	0.07976 (10)	0.19299 (9)	0.86268 (10)	0.0183 (2)	
H14C	0.1449	0.2214	0.8757	0.022*	
H14D	0.0370	0.2026	0.9164	0.022*	
H1NB	−0.0909 (14)	0.1209 (13)	0.8571 (13)	0.026 (5)*	
H1NA	0.8714 (15)	0.0766 (14)	0.3890 (14)	0.033 (5)*	

### Atomic displacement parameters (Å^2^)

**Table:**

	*U*^11^	*U*^22^	*U*^33^	*U*^12^	*U*^13^	*U*^23^
Br1C	0.0235 (14)	0.0151 (5)	0.0216 (5)	0.0021 (5)	−0.0005 (4)	0.0042 (4)
Br1A	0.0622 (9)	0.0206 (5)	0.0219 (3)	0.0145 (5)	−0.0070 (5)	0.0008 (3)
O1A	0.0180 (5)	0.0153 (5)	0.0366 (6)	0.0002 (4)	−0.0001 (4)	0.0009 (4)
O2A	0.0153 (5)	0.0257 (6)	0.0385 (6)	0.0054 (4)	0.0024 (4)	0.0008 (5)
O3A	0.0225 (5)	0.0295 (6)	0.0319 (6)	−0.0083 (4)	0.0058 (4)	−0.0021 (5)
O4A	0.0369 (6)	0.0174 (5)	0.0295 (5)	−0.0069 (4)	0.0016 (5)	0.0009 (4)
N1A	0.0132 (5)	0.0166 (5)	0.0183 (5)	0.0016 (4)	0.0011 (4)	0.0005 (4)
N2A	0.0151 (5)	0.0134 (5)	0.0223 (5)	0.0013 (4)	0.0014 (4)	0.0004 (4)
N3A	0.0170 (5)	0.0175 (5)	0.0206 (5)	0.0021 (4)	−0.0008 (4)	−0.0011 (4)
N4A	0.0263 (6)	0.0212 (6)	0.0169 (5)	−0.0075 (5)	0.0036 (4)	−0.0019 (4)
C1A	0.0192 (6)	0.0157 (6)	0.0196 (6)	−0.0024 (5)	0.0011 (5)	0.0004 (5)
C2A	0.0166 (6)	0.0243 (7)	0.0215 (6)	−0.0056 (5)	0.0022 (5)	0.0000 (5)
C3A	0.0157 (6)	0.0265 (7)	0.0210 (6)	0.0021 (5)	0.0016 (5)	−0.0002 (5)
C4A	0.0194 (6)	0.0180 (6)	0.0237 (6)	0.0037 (5)	0.0005 (5)	0.0001 (5)
C5A	0.0166 (6)	0.0153 (6)	0.0212 (6)	−0.0008 (5)	0.0009 (5)	0.0007 (5)
C6A	0.0148 (6)	0.0148 (6)	0.0150 (5)	−0.0004 (4)	−0.0006 (4)	−0.0003 (4)
C7A	0.0165 (6)	0.0138 (6)	0.0160 (5)	0.0013 (4)	−0.0002 (4)	0.0002 (4)
C8A	0.0153 (6)	0.0158 (6)	0.0155 (5)	0.0000 (4)	−0.0001 (4)	−0.0003 (4)
C9A	0.0174 (6)	0.0165 (6)	0.0176 (5)	0.0024 (5)	0.0024 (4)	−0.0011 (4)
C10A	0.0240 (6)	0.0153 (6)	0.0172 (6)	−0.0004 (5)	0.0013 (5)	−0.0009 (4)
C11A	0.0210 (6)	0.0171 (6)	0.0156 (5)	−0.0044 (5)	0.0020 (5)	−0.0007 (4)
C12A	0.0162 (6)	0.0208 (6)	0.0161 (5)	−0.0016 (5)	0.0002 (4)	−0.0013 (5)
C13A	0.0166 (6)	0.0155 (6)	0.0169 (5)	0.0011 (5)	0.0005 (4)	−0.0004 (4)
C14A	0.0190 (6)	0.0161 (6)	0.0186 (6)	0.0011 (5)	−0.0003 (5)	−0.0003 (4)
Br1B	0.02671 (7)	0.01604 (7)	0.02483 (7)	0.00470 (5)	0.00414 (5)	0.00186 (5)
O1B	0.0200 (5)	0.0146 (5)	0.0345 (6)	0.0001 (4)	0.0014 (4)	0.0018 (4)
O2B	0.0189 (5)	0.0243 (6)	0.0738 (9)	0.0085 (5)	0.0131 (6)	0.0068 (6)
O3B	0.0159 (5)	0.0297 (6)	0.0446 (7)	−0.0018 (4)	0.0024 (4)	0.0081 (5)
O4B	0.0277 (6)	0.0174 (5)	0.0410 (7)	−0.0017 (4)	0.0071 (5)	0.0046 (4)
N1B	0.0132 (5)	0.0160 (5)	0.0200 (5)	0.0013 (4)	0.0017 (4)	−0.0007 (4)
N2B	0.0137 (5)	0.0134 (5)	0.0245 (5)	0.0016 (4)	0.0025 (4)	−0.0003 (4)
N3B	0.0174 (5)	0.0158 (5)	0.0272 (6)	0.0027 (4)	0.0011 (4)	−0.0003 (4)
N4B	0.0190 (5)	0.0214 (6)	0.0197 (5)	−0.0031 (4)	0.0022 (4)	0.0011 (4)
C1B	0.0182 (6)	0.0151 (6)	0.0211 (6)	−0.0017 (5)	0.0024 (5)	−0.0008 (5)
C2B	0.0154 (6)	0.0209 (7)	0.0243 (6)	−0.0029 (5)	0.0030 (5)	−0.0009 (5)
C3B	0.0158 (6)	0.0228 (7)	0.0220 (6)	0.0035 (5)	0.0020 (5)	0.0010 (5)
C4B	0.0191 (6)	0.0162 (6)	0.0280 (7)	0.0028 (5)	0.0029 (5)	0.0019 (5)
C5B	0.0167 (6)	0.0155 (6)	0.0247 (6)	−0.0011 (5)	0.0029 (5)	0.0009 (5)
C6B	0.0149 (6)	0.0148 (6)	0.0152 (5)	−0.0001 (4)	0.0006 (4)	−0.0011 (4)
C7B	0.0160 (6)	0.0135 (6)	0.0163 (5)	0.0003 (4)	0.0009 (4)	−0.0013 (4)
C8B	0.0142 (6)	0.0154 (6)	0.0157 (5)	0.0007 (4)	0.0006 (4)	−0.0015 (4)
C9B	0.0152 (6)	0.0153 (6)	0.0202 (6)	0.0024 (5)	0.0009 (4)	−0.0002 (4)
C10B	0.0189 (6)	0.0148 (6)	0.0192 (6)	0.0015 (5)	0.0014 (5)	0.0001 (4)
C11B	0.0150 (6)	0.0179 (6)	0.0167 (5)	−0.0015 (5)	0.0006 (4)	0.0014 (4)
C12B	0.0141 (6)	0.0189 (6)	0.0179 (6)	0.0008 (5)	0.0012 (4)	−0.0003 (4)
C13B	0.0145 (6)	0.0143 (6)	0.0186 (6)	0.0023 (4)	0.0003 (4)	−0.0005 (4)
C14B	0.0179 (6)	0.0150 (6)	0.0221 (6)	0.0003 (5)	0.0014 (5)	−0.0023 (5)

### Geometric parameters (Å, °)

**Table:**

Br1C—C14A	1.949 (4)	C14A—H14F	0.9601
Br1A—C14A	1.962 (3)	Br1B—C14B	1.9699 (14)
O1A—N3A	1.2413 (15)	O1B—N3B	1.2388 (16)
O2A—N3A	1.2265 (15)	O2B—N3B	1.2232 (16)
O3A—N4A	1.2286 (17)	O3B—N4B	1.2252 (16)
O4A—N4A	1.2321 (17)	O4B—N4B	1.2347 (16)
N1A—C7A	1.2955 (17)	N1B—C7B	1.2922 (17)
N1A—N2A	1.3636 (16)	N1B—N2B	1.3604 (16)
N2A—C8A	1.3572 (17)	N2B—C8B	1.3584 (17)
N2A—H1NA	0.79 (2)	N2B—H1NB	0.82 (2)
N3A—C13A	1.4487 (17)	N3B—C13B	1.4486 (17)
N4A—C11A	1.4549 (18)	N4B—C11B	1.4559 (18)
C1A—C2A	1.392 (2)	C1B—C2B	1.3928 (19)
C1A—C6A	1.4010 (18)	C1B—C6B	1.3995 (18)
C1A—H1AA	0.9300	C1B—H1BA	0.9300
C2A—C3A	1.386 (2)	C2B—C3B	1.389 (2)
C2A—H2AA	0.9300	C2B—H2BA	0.9300
C3A—C4A	1.392 (2)	C3B—C4B	1.390 (2)
C3A—H3AA	0.9300	C3B—H3BA	0.9300
C4A—C5A	1.3847 (19)	C4B—C5B	1.382 (2)
C4A—H4AA	0.9300	C4B—H4BA	0.9300
C5A—C6A	1.4031 (19)	C5B—C6B	1.4011 (19)
C5A—H5AA	0.9300	C5B—H5BA	0.9300
C6A—C7A	1.4816 (18)	C6B—C7B	1.4824 (18)
C7A—C14A	1.4973 (18)	C7B—C14B	1.4969 (18)
C8A—C9A	1.4155 (19)	C8B—C13B	1.4164 (18)
C8A—C13A	1.4205 (19)	C8B—C9B	1.4185 (18)
C9A—C10A	1.3727 (19)	C9B—C10B	1.3706 (19)
C9A—H9AA	0.9300	C9B—H9BA	0.9300
C10A—C11A	1.396 (2)	C10B—C11B	1.3980 (19)
C10A—H10A	0.9300	C10B—H10B	0.9300
C11A—C12A	1.375 (2)	C11B—C12B	1.3714 (19)
C12A—C13A	1.3851 (19)	C12B—C13B	1.3928 (19)
C12A—H12A	0.9300	C12B—H12B	0.9300
C14A—H14A	0.9600	C14B—H14C	0.9700
C14A—H14B	0.9600	C14B—H14D	0.9700
C14A—H14E	0.9600		
			
C7A—N1A—N2A	117.26 (11)	H14A—C14A—H14E	104.4
C8A—N2A—N1A	118.61 (11)	C7A—C14A—H14F	109.8
C8A—N2A—H1NA	118.8 (15)	Br1C—C14A—H14F	106.0
N1A—N2A—H1NA	122.1 (15)	Br1A—C14A—H14F	109.9
O2A—N3A—O1A	122.50 (12)	H14B—C14A—H14F	112.0
O2A—N3A—C13A	118.70 (12)	H14E—C14A—H14F	108.2
O1A—N3A—C13A	118.80 (11)	C7B—N1B—N2B	117.50 (11)
O3A—N4A—O4A	123.35 (13)	C8B—N2B—N1B	118.85 (11)
O3A—N4A—C11A	118.47 (13)	C8B—N2B—H1NB	115.9 (13)
O4A—N4A—C11A	118.17 (12)	N1B—N2B—H1NB	124.8 (14)
C2A—C1A—C6A	120.43 (13)	O2B—N3B—O1B	122.01 (12)
C2A—C1A—H1AA	119.8	O2B—N3B—C13B	118.52 (12)
C6A—C1A—H1AA	119.8	O1B—N3B—C13B	119.46 (11)
C3A—C2A—C1A	120.29 (13)	O3B—N4B—O4B	123.33 (13)
C3A—C2A—H2AA	119.9	O3B—N4B—C11B	118.80 (12)
C1A—C2A—H2AA	119.9	O4B—N4B—C11B	117.86 (12)
C2A—C3A—C4A	119.77 (13)	C2B—C1B—C6B	120.41 (13)
C2A—C3A—H3AA	120.1	C2B—C1B—H1BA	119.8
C4A—C3A—H3AA	120.1	C6B—C1B—H1BA	119.8
C5A—C4A—C3A	120.31 (13)	C3B—C2B—C1B	120.36 (13)
C5A—C4A—H4AA	119.8	C3B—C2B—H2BA	119.8
C3A—C4A—H4AA	119.8	C1B—C2B—H2BA	119.8
C4A—C5A—C6A	120.56 (13)	C2B—C3B—C4B	119.46 (13)
C4A—C5A—H5AA	119.7	C2B—C3B—H3BA	120.3
C6A—C5A—H5AA	119.7	C4B—C3B—H3BA	120.3
C1A—C6A—C5A	118.62 (12)	C5B—C4B—C3B	120.46 (13)
C1A—C6A—C7A	121.50 (12)	C5B—C4B—H4BA	119.8
C5A—C6A—C7A	119.87 (12)	C3B—C4B—H4BA	119.8
N1A—C7A—C6A	116.21 (11)	C4B—C5B—C6B	120.79 (13)
N1A—C7A—C14A	123.25 (12)	C4B—C5B—H5BA	119.6
C6A—C7A—C14A	120.54 (12)	C6B—C5B—H5BA	119.6
N2A—C8A—C9A	120.14 (12)	C1B—C6B—C5B	118.52 (12)
N2A—C8A—C13A	122.97 (12)	C1B—C6B—C7B	122.27 (12)
C9A—C8A—C13A	116.88 (12)	C5B—C6B—C7B	119.20 (12)
C10A—C9A—C8A	121.67 (13)	N1B—C7B—C6B	116.03 (12)
C10A—C9A—H9AA	119.2	N1B—C7B—C14B	122.85 (12)
C8A—C9A—H9AA	119.2	C6B—C7B—C14B	120.95 (11)
C9A—C10A—C11A	119.21 (13)	N2B—C8B—C13B	122.72 (12)
C9A—C10A—H10A	120.4	N2B—C8B—C9B	120.22 (12)
C11A—C10A—H10A	120.4	C13B—C8B—C9B	117.05 (12)
C12A—C11A—C10A	121.58 (13)	C10B—C9B—C8B	121.37 (12)
C12A—C11A—N4A	118.77 (13)	C10B—C9B—H9BA	119.3
C10A—C11A—N4A	119.64 (13)	C8B—C9B—H9BA	119.3
C11A—C12A—C13A	119.15 (13)	C9B—C10B—C11B	119.39 (12)
C11A—C12A—H12A	120.4	C9B—C10B—H10B	120.3
C13A—C12A—H12A	120.4	C11B—C10B—H10B	120.3
C12A—C13A—C8A	121.50 (12)	C12B—C11B—C10B	121.76 (13)
C12A—C13A—N3A	116.18 (12)	C12B—C11B—N4B	119.05 (12)
C8A—C13A—N3A	122.30 (12)	C10B—C11B—N4B	119.18 (12)
C7A—C14A—Br1C	109.55 (14)	C11B—C12B—C13B	118.76 (12)
C7A—C14A—Br1A	109.50 (11)	C11B—C12B—H12B	120.6
C7A—C14A—H14A	109.8	C13B—C12B—H12B	120.6
Br1C—C14A—H14A	109.8	C12B—C13B—C8B	121.62 (12)
Br1A—C14A—H14A	113.6	C12B—C13B—N3B	116.26 (12)
C7A—C14A—H14B	109.8	C8B—C13B—N3B	122.11 (12)
Br1C—C14A—H14B	109.7	C7B—C14B—Br1B	111.51 (9)
Br1A—C14A—H14B	105.8	C7B—C14B—H14C	109.3
H14A—C14A—H14B	108.2	Br1B—C14B—H14C	109.3
C7A—C14A—H14E	109.8	C7B—C14B—H14D	109.3
Br1C—C14A—H14E	113.5	Br1B—C14B—H14D	109.3
Br1A—C14A—H14E	109.7	H14C—C14B—H14D	108.0
			
C7A—N1A—N2A—C8A	−176.60 (12)	C6A—C7A—C14A—Br1A	98.2 (2)
C6A—C1A—C2A—C3A	0.5 (2)	C7B—N1B—N2B—C8B	−170.40 (12)
C1A—C2A—C3A—C4A	−0.1 (2)	C6B—C1B—C2B—C3B	0.6 (2)
C2A—C3A—C4A—C5A	0.0 (2)	C1B—C2B—C3B—C4B	0.1 (2)
C3A—C4A—C5A—C6A	−0.3 (2)	C2B—C3B—C4B—C5B	−0.6 (2)
C2A—C1A—C6A—C5A	−0.80 (19)	C3B—C4B—C5B—C6B	0.4 (2)
C2A—C1A—C6A—C7A	178.94 (12)	C2B—C1B—C6B—C5B	−0.80 (19)
C4A—C5A—C6A—C1A	0.70 (19)	C2B—C1B—C6B—C7B	177.96 (12)
C4A—C5A—C6A—C7A	−179.04 (12)	C4B—C5B—C6B—C1B	0.3 (2)
N2A—N1A—C7A—C6A	−177.18 (11)	C4B—C5B—C6B—C7B	−178.51 (13)
N2A—N1A—C7A—C14A	3.11 (18)	N2B—N1B—C7B—C6B	−179.43 (11)
C1A—C6A—C7A—N1A	170.46 (12)	N2B—N1B—C7B—C14B	5.26 (19)
C5A—C6A—C7A—N1A	−9.80 (18)	C1B—C6B—C7B—N1B	177.49 (12)
C1A—C6A—C7A—C14A	−9.82 (18)	C5B—C6B—C7B—N1B	−3.76 (18)
C5A—C6A—C7A—C14A	169.92 (12)	C1B—C6B—C7B—C14B	−7.11 (19)
N1A—N2A—C8A—C9A	4.05 (18)	C5B—C6B—C7B—C14B	171.65 (12)
N1A—N2A—C8A—C13A	−176.93 (12)	N1B—N2B—C8B—C13B	−178.28 (12)
N2A—C8A—C9A—C10A	179.39 (12)	N1B—N2B—C8B—C9B	2.88 (18)
C13A—C8A—C9A—C10A	0.31 (19)	N2B—C8B—C9B—C10B	−179.15 (12)
C8A—C9A—C10A—C11A	0.0 (2)	C13B—C8B—C9B—C10B	1.94 (19)
C9A—C10A—C11A—C12A	−0.7 (2)	C8B—C9B—C10B—C11B	−1.1 (2)
C9A—C10A—C11A—N4A	178.16 (12)	C9B—C10B—C11B—C12B	−0.9 (2)
O3A—N4A—C11A—C12A	6.17 (18)	C9B—C10B—C11B—N4B	179.49 (12)
O4A—N4A—C11A—C12A	−174.63 (12)	O3B—N4B—C11B—C12B	6.41 (19)
O3A—N4A—C11A—C10A	−172.69 (12)	O4B—N4B—C11B—C12B	−174.33 (13)
O4A—N4A—C11A—C10A	6.51 (18)	O3B—N4B—C11B—C10B	−173.95 (13)
C10A—C11A—C12A—C13A	1.1 (2)	O4B—N4B—C11B—C10B	5.31 (18)
N4A—C11A—C12A—C13A	−177.78 (11)	C10B—C11B—C12B—C13B	1.9 (2)
C11A—C12A—C13A—C8A	−0.76 (19)	N4B—C11B—C12B—C13B	−178.46 (12)
C11A—C12A—C13A—N3A	177.91 (11)	C11B—C12B—C13B—C8B	−0.99 (19)
N2A—C8A—C13A—C12A	−178.96 (12)	C11B—C12B—C13B—N3B	178.53 (12)
C9A—C8A—C13A—C12A	0.09 (19)	N2B—C8B—C13B—C12B	−179.76 (12)
N2A—C8A—C13A—N3A	2.5 (2)	C9B—C8B—C13B—C12B	−0.88 (19)
C9A—C8A—C13A—N3A	−178.49 (11)	N2B—C8B—C13B—N3B	0.7 (2)
O2A—N3A—C13A—C12A	−0.45 (18)	C9B—C8B—C13B—N3B	179.62 (12)
O1A—N3A—C13A—C12A	−179.62 (12)	O2B—N3B—C13B—C12B	5.65 (19)
O2A—N3A—C13A—C8A	178.20 (12)	O1B—N3B—C13B—C12B	−173.60 (12)
O1A—N3A—C13A—C8A	−0.97 (19)	O2B—N3B—C13B—C8B	−174.83 (14)
N1A—C7A—C14A—Br1C	−77.37 (17)	O1B—N3B—C13B—C8B	5.92 (19)
C6A—C7A—C14A—Br1C	102.93 (15)	N1B—C7B—C14B—Br1B	−77.67 (14)
N1A—C7A—C14A—Br1A	−82.1 (2)	C6B—C7B—C14B—Br1B	107.25 (12)

### Hydrogen-bond geometry (Å, °)

**Table:**

*D*—H···*A*	*D*—H	H···*A*	*D*···*A*	*D*—H···*A*
N2B—H1NB···Br1B	0.82 (2)	2.826 (19)	3.3764 (12)	126.2 (15)
N2B—H1NB···O1B	0.82 (2)	1.969 (19)	2.6159 (16)	135.1 (17)
N2A—H1NA···O1A	0.79 (2)	2.02 (2)	2.6120 (16)	131.8 (19)
C2B—H2BA···Br1A^i^	0.93	2.93	3.673 (3)	138
C14B—H14C···O1A^i^	0.97	2.49	3.3352 (17)	145
C14B—H14D···O3A^ii^	0.97	2.52	3.3745 (18)	147

Symmetry codes: (i) *x*−1/2, −*y*+1/2, *z*+1/2; (ii) −*x*+1/2, *y*+1/2, −*z*+3/2.
